# 1007. Case Study. Burn Wound Management with Lyophilized Acellular Pisces Dermis at an Underserved South Pacific Island

**DOI:** 10.1093/jbcr/irag033.195

**Published:** 2026-04-06

**Authors:** Alfredo C Cordova, Kristy Miller, Talia Selembo, Victoria Young

**Affiliations:** Sarasota Memorial Hospital, Sarasota, FL; Sarasota Memorial Hospital, Sarasota, FL; Sarasota Memorial Hospital, Sarasota, FL; Sarasota Memorial Hospital, Sarasota, FL

## Abstract

**Patient Presentation (age range, injury details, relevant history):**

Twenty-four patients with different wound etiologies were treated with lyophilized fish dermis during a military humanitarian mission at the Polynesian island of American Samoa. This included 2 burn patients from scald injuries, with mixed depth, and with multiple comorbidities including stroke, diabetes mellitus, peripheral vascular disease, obesity, and gout.

**Clinical Challenges:**

The South Pacific islands may be particularly vulnerable due to the limited access to medical care. Wounds of different etiologies, including burn injuries, can be challenging to treat even worse given their limited resources. Dermal matrices may enhance the development of an optimal wound bed for grafting, provide temporary wound coverage, and decrease the healing time. Decellularized and lyophilized fish dermis have proven beneficial effects in the 4-stages of wound healing. Its application may allow an accelerated and complete healing of these wounds.

**Management Approach:**

Body parts affected with scald burns included the perineum, genitalia, the upper and lower extremities. Tangential excisional debridement was performed. Subsequently, they were resurfaced with fish dermis and wall suction negative pressure therapy. No autologous skin grafting was necessary.

**Outcomes:**

Graft incorporation, granulation tissue formation, and neovascularization was evidenced in >95% of the surface area in all cases by 10-14 days after xenograft application. Skin coverage with meshed STSG was not necessary. Wounds were allowed to heal by secondary intention with complete healing within 21-25 days.

**Lessons Learned:**

Decellularized and lyophilized fish dermis provide excellent wound coverage and accelerates healing on mixed depth wounds avoiding autologous grafting as observed in these South Pacific islanders. This may be reproduced in other underserved populations.

